# Effects of nitrogen addition and *Bothriochloa ischaemum* and *Lespedeza davurica* mixture on plant chlorophyll fluorescence and community production in semi-arid grassland

**DOI:** 10.3389/fpls.2024.1400309

**Published:** 2024-06-24

**Authors:** Fugang Wang, Lei Shi, Ruiyi Zhang, Weizhou Xu, Yaojun Bo

**Affiliations:** ^1^ College of Life Science, Yulin University, Yulin, China; ^2^ Shaanxi Engineering Research Center of Forage Plants of the Loess Plateau, Yulin University, Yulin, Shaanxi, China

**Keywords:** grass-legume mixture, nitrogen addition, chlorophyll fluorescence, aboveground biomass, overyielding

## Abstract

**Background:**

Grass-legume mixture can effectively improve productivity and stimulate overyielding in artificial grasslands, but may be N-limited in semi-arid regions. This study investigated the effects of N addition on chlorophyll fluorescence and production in the grass-legume mixtures community.

**Methods:**

An N addition experiment was conducted in the *Bothriochloa ischaemum* and *Lespedeza davurica* mixture community, with seven mixture ratios (B0L10, B2L8, B4L6, B5L5, B6L4, B8L2, and B10L0) according to the sowing abundance of *B.ischaemum* and *L.davurica* and four N addition levels, N0, N25, N50, and N75 (0,25,50,75kgNhm^-2^ a^-1^), respectively. We analyzed the response of chlorophyll fluorescence parameters of the two species, the rapid light-response curves of chlorophyll fluorescence, as well as aboveground biomass (AGB) and overyielding.

**Results:**

Our results showed that the two species showed different photosynthetic strategies, with *L.davurica* having significantly higher initial fluorescence (Fo), effective photochemical quantum yield of PSII (ΦPSII), and coefficient of photochemical fluorescence quenching (qP) than *B. ischaemum*, consisting with results of rapid light-response curves. N addition and mixture ratio both had significant effects on chlorophyll fluorescence and AGB (*p*<0.001). The ΦPSII and qP of *L.davurica* were significantly lowest in B5L5 and B6L4 under N addition, and the effect of N varied with mixture ratio. The photosynthetic efficiency of *B. ischaemum* was higher in mixture than in monoculture (B10L0), and ΦPSII was significantly higher in N50 than in N25 and N50 at mixture communities except at B5L5. The community AGB was significantly higher in mixture communities than in two monocultures and highest at B6L4. In the same mixture ratio, the AGB was highest under the N50. The overyielding effects were significantly highest under the N75 and B6L4 treatments, mainly attributed to *L.davurica.* The partial least squares path models demonstrated that adding N increased soil nutrient content, and complementary utilization by *B.ischaemum* and *L.davurica* increased the photosynthetic efficiency. However, as the different photosynthetic strategies of these two species, the effect on AGB was offset, and the mixture ratio’s effects were larger than N. Our results proposed the B6L4 and N50 treatments were the optimal combination, with the highest AGB and overyielding, moderate grass-legume ratio, optimal community structure, and forage values.

## 1 Introduction

Mixing species with complementary functional traits in time and/or space is a widely adopted planting pattern for artificial grassland (Schipanski and Drinkwater, 2012). Mixture sowing can increase resource use efficiency and complementarity, thereby enhancing biomass and ecosystem function (De Long et al., 2019). The grass-legume mixtures is a widely used model for obtaining high yields and forage value, also improve grassland diversity and stability (Yan et al., 2022). It is well known that legumes have rhizobia that fixes nitrogen (N_2_) in the atmosphere, thus easing nitrogen competition interspecies (Schipanski and Drinkwater, 2012). Generally speaking, symbiosis with legume species can increase soil nitrogen pool, enhance nitrogen transformation rate, and reduce nutrient limitation to some extent (Wang et al., 2022). Plant relations can be positive (facilitation, N_2_ fixation), but inappropriate species combinations can also be negative (competitive exclusion, allelopathy) (Sanderson et al., 2004). Consequently, mixing in grassland ecosystems needs to consider the interspecific connections and species’ adaptations to their environments. The *Bothriochloa ischaemum* is a C4 perennial herbaceous plant, with excellent characteristics, such as strong tillering power, drought resistance, trampling resistance, strong soil and water retention, etc. The *Lespedeza davurica* is a C3 leguminous plant, with strong adaptability and wide distribution, and an excellent native forage grasses that livestock prefer. For a long time, *B. ischaemum* and *L. davurica* have had a good symbiotic relationship in temperate typical grasslands, which have considerable conservational and agricultural values. In the field experiment of *B. ischaemum* and *L. davurica* mixture, *B. ischaemum* had higher photosynthesis and leaf water use efficiency than *L. davurica* at B8D2 and B6D4 ratios (Wang et al., 2012). Previous pot experiment studies suggest that the 8:4 ratio of *B. ischaemum* and *L. davurica* mixture had significantly greater relative yield totals, transpiration water use efficiency, and biomass production under deficit water conditions (Xu et al., 2011). Furthermore, the N and P capture and absorption of *B. ischaemum* were greatly improved by both fertilization and the mixture proportions (Xu et al., 2016). This distinct evidence suggests that the optimal mixture ratio of artificial grasslands of *B. ischaemum* and *L. davurica* remains controversial. Studying species’ photosynthetic physiology, interspecific competition and complementarity, and nutrient utilization strategies can contribute to reasonable grassland structure, high yield, and high community stability in grass-legume mixtures, with significant ecological and practical implications (Xu et al., 2011, 2016).

The grass-legume mixtures is in the pursuit of higher productivity, and a reasonable mixture ratio is one of the effective ways to improve the utilization of light energy (Liu et al., 2016). The hypotheses of interspecies relationship, material cycle, and soil ecology can explain the overyielding effect in a mixture community (Li et al., 2014; Van der Werf et al., 2021). Li et al. (2015) found that soil total nitrogen and available nitrogen concentrations increased with legume component ratios (from 1:0 to 1:1 of grass: legume), but further decreased when the grass: legume ratio was 1:3 to 0:1 (legume monoculture). Legume introduction can serve as an alternative to nitrogen fertilization for enhancing grassland productivity (Li et al., 2015). One study in European grassland showed that the total yield of mixture cultivation exceeded that of monoculture by more than 97%, and the invasion of forbs weakened the production of legumes with the extension of time (Finn et al., 2013). Yan et al. analyzed the relationship between grass-legume mixtures and forage production through bacterial community and pointed out that the rhizosphere bacterial community played a critical role in enhancing plant growth (Yan et al., 2022). However, although nitrogen (N) addition in mixture community did not significantly affect soil nutrients and microbial biomass, the production of mixture communities was higher than that of monocropping communities (Dhakal and Anowarul Islam, 2018). The mixture had higher net photosynthesis, water use efficiency, and leaf nitrogen content, lower carbon-to-nitrogen ratios, and absorbed light that was used more for photosynthetic electron transfer and heat dissipation (Liu et al., 2016). When the responses of species to the environment are not perfectly positively correlated, declines in some populations are compensated by increases in others, thus diminishing the variability of ecosystem production (Hector et al., 2010). Inevitably, there exists intensive competition or synergistic interaction among species, and the differences in photosynthetic traits of species affect the photosynthetic capacity of the community, and ultimately the production of the community (Schreiber et al., 1995; Finn et al., 2013).

The advantage of the grass-legume mixture lies in the complementary utilization of nutrients and light resources between the two species. Light is the key factor in synthesizing organic matter. Chlorophyll fluorescence parameters and rapid light-response curves reflect the light energy conversion efficiency, electron transfer rate, energy dissipation rate, and light response characteristics of plant photosystem II (Schreiber et al., 1995; White and Critchley, 1999; Baker and Rosenqvist, 2004). Therefore, chlorophyll fluorescence is more sensitive in detecting plant adaptations in response to environmental changes and understanding the photosynthetic strategies of different species (Frankenberg and Berry, 2017). Over the past century, the effects of drought stress (Zhao et al., 2019), nutrient addition (Lin et al., 2013; Yue et al., 2023), and shading (Hallik et al., 2012) on chlorophyll fluorescence have been a research hotspot, and the differential responses in different studies are closely linked to the genetic characteristics of species, habitat characteristics (Sharma et al., 2020). Frankow-Lindberg et al, confirmed that legumes were able to transfer 17.08 kg N ha^-1^ to non-legumes in the community, indirectly promoting photosynthesis in other species (Frankow-Lindberg and Dahlin, 2013). Current studies mainly focus on the influences of habitats or environmental stress, still lacking an understanding of how interspecific symbiotic or competitive relationships affect species’ chlorophyll fluorescence in the mixture community.

Soil nitrogen content in semi-arid grasslands is relatively low due to external factors such as soil erosion and intensified anthropogenic activities, which limits plant growth on the Loess Plateau (Jiao et al., 2011). N addition is an effective way to boost production and improve community structure, mainly by increasing the nitrogen content of leaves, chlorophyll content photosynthetic enzyme activity, etc. to enhance the efficiency of plant photosynthesis (Chen et al., 2020a). Lin et al. demonstrated that N application increased the maximum photochemical efficiency (Fv/Fm), effective photochemical quantum yield of PSII (ΦPS II), electron transfer rate (ETR), and coefficient of photochemical fluorescence quenching (qP) of photosystem II (PSII) of naked oat (*Avena nuda L.*) (Lin et al., 2013). After N addition, the maximum quantum yield of photosystem II was correlated with both basic and supplemental N application of spring triticale (Janušauskaite and Feiziene, 2012). Research conducted in the semi-arid Loess Plateau revealed that N and P addition markedly boosted the activity of photosystem II (ΦPSII) of *B.ischaemum* and *L.davurica*. Additionally, the response degree was regulated by species attributes and fertilization levels (Lai et al., 2021). Ullah et al. discovered that high N supply increased photochemical efficiency (Fv/Fm) but failed to promote biomass accumulation, emphasizing the importance of nutrient level (Ullah et al., 2020). In summary, mixture affects the photosynthetic strategy of plants by changing interspecific relationships within the community, and there also exists species-specificity response of plant chlorophyll fluorescence to N addition.

We are interested in whether the combination of mixture sowing and exogenous nitrogen addition can further enhance plant photosynthetic capacity and how it will affect the productivity of the community. Based on the above background, the importance and originality of this study was to investigate the effects of N addition on the chlorophyll fluorescence parameters and production in the *B.ischaemum* and *L.davurica* mixture community. Specifically, we consider three questions: (1) Effect of mixture and N interaction on the chlorophyll fluorescence characteristics on *B.ischaemum* and *L.davurica*. (2) The effect of mixture and N interaction on community production and whether it produces an overyielding effect. (3) Associations between plant chlorophyll fluorescence and community production of *B.ischaemum* and *L.davurica*. This study aimed to investigate the physiological and ecological effects of N addition and mixture sowing on the vegetation of the Loess Plateau, and to provide a scientific basis for vegetation restoration and management on the Loess Plateau.

## 2 Materials and methods

### 2.1 Experimental site description

The study area is located in Ansai County, Shaanxi Province, with a geographic location of E109°19’23″, N36°51’30″, and an average elevation of 1100–1300 m. The annual mean temperature is 8.8°C, and the annual precipitation is 541 mm. The rainfall distribution is uneven across seasons, with 60% to 80% of the annual rainfall occurring from July to September. The climate type is transitional warm-temperate semi-arid, with vegetation type transit from warm temperate deciduous broad-leaved forest to steppe. The zonal herbaceous vegetation includes *B.ischaemum*, *L.davurica*, *Artemisia sacrorum*, *Thymus mongolicus*, *Stipa grandis*, *Stipa bungeana*, etc.

### 2.2 Experimental design

**Figure 1:**
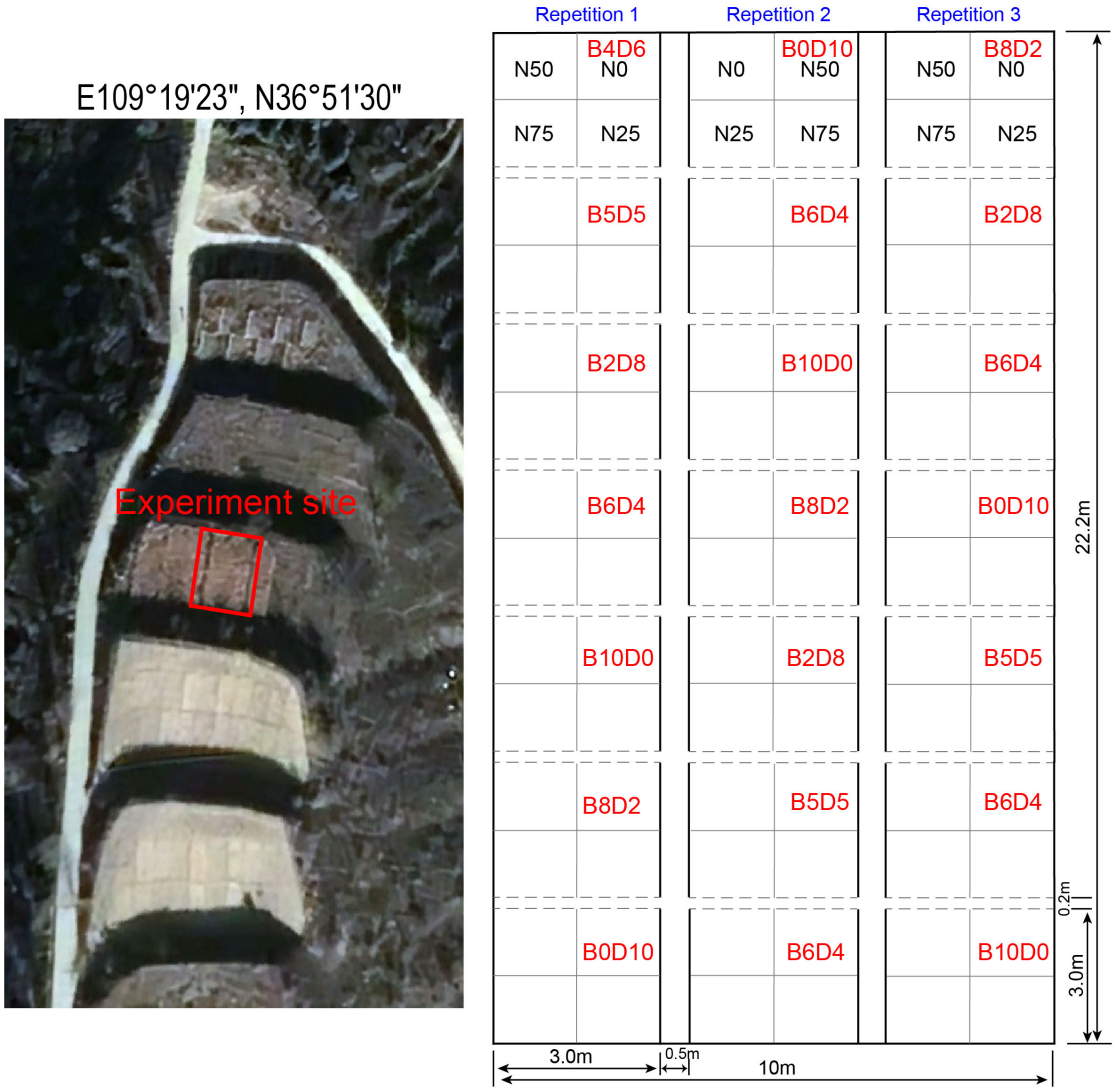
Experiment site location and experimental design schematic.

The experiment site is in the mountainous experimental terraced field of ‘Ansai Soil and Water Conservation Comprehensive Experimental Station of the Chinese Academy of Sciences, with 23 m long and 10 m wide (**Figure 1**). The grass-legume mixture experiment started in July 2009, using two typical native species, *B.ischaemum* and *L.davurica* (Wang et al., 2012). The mixture ratio was designed using the ecological substitution method, that is, the total number of sowing densities remained constant, but changed the sowing ratio of these two species. Seven combinations of mixture ratios (i.e., B0L10, B2L8, B4L6, B5L5, B6L4, B8L2, and B10L0) were set according to the sowing abundance of *B.ischaemum* and *L.davurica*. Seven main plots with the seven mixing ratios were arranged vertically for each repetition, and three repetitions were spaced apart using a 50 cm buffer. All seven mixture ratios were set by a completely randomized block design in each repetition. There were 21 main plots with seven mixing ratios in total. Each main plot was 3m×3m, and the row spacing was 20cm. No fertilization or irrigation was applied during the test period, and other forbs were removed at the right time in the middle of each month during the growing season, at last, all plants’ aboveground parts were mowed flush at the end of the reproductive period at the end of each year.

In August 2018, we conducted a basic survey on the mixture experimental field and measured the soil total nitrogen, and soil total phosphorus contents. In 2019, the second stage N addition experiment started in May 2019, before the growing season. The N addition experiment was carried out in the mixture-sowing site. The main plot of seven mixture ratios was divided into four subplots (1.5 m ×1.5 m) within each 3m × 3m plot. Four N levels were applied: N0 (0 kg N hm^-2^ a^-1^, blank control), N25 (25 kg N hm^-2^ a^-1^), N50 (50 kg N hm^-2^ a^-1^), and N75 (75 kg N hm^-2^ a^-1^) (Chen et al., 2020b). Unlike the mixture treatments, N addition was distributed consistently across the same replicate, but another distribution order was used in another replicate (**Figure 1**). Combining the mixture ratio and N addition, there were 28 treatments in total, with three replicates for each treatment, resulting in 84 plots.

### 2.3 Determination of chlorophyll fluorescence parameters and rapid light-response curves

The chlorophyll fluorescence parameters were measured using PAM-2500 (Walz Company) on 11–13 August 2019 from 8:00 a.m. to 12:00 p.m. At this time, *B.ischaemum* was in the tassel stage and *L.davurica* was in the flowering stage. In each subplot, three plants of *B. ischaemum* and three plants of *L.davurica* were randomly selected, and a fully expanded mature leaf for determination. Measurements were averaged over the three plants. Leaf clamps were opened after 30 minutes of dark adaptation, and the following chlorophyll fluorescence parameters were automatically calculated by the system in the selected mode, including initial fluorescence (Fo), Maximum quantum efficiency of PSII photochemistry (Fv/Fm), effective photochemical quantum yield of PSII (ΦPSII), non-photochem-ical fluorescence quenching (NPQ), coefficient of photochemical fluorescence quenching (qP) (Baker, 2008).

Next, the rapid light-response curves of chlorophyll fluorescence were conducted to analyze the two species’ photosynthetic performance (electron transport rate, ETR) with changing photosynthetically active radiation (PAR) (Baker, 2008). ETR is the electron transport rate, which is an important indicator of the light energy conversion efficiency of photosystem II. The rapid light-response curves of chlorophyll fluorescence were measured using the multiphase pulse technique by PAM-2500. In each sample plot, fully expanded leaves of *B. ischaemum* and *L.davurica* were randomly selected, and the leaf chamber leaves were exposed to different photosynthetically active radiation (PAR) gradients [0, 60, 150, 250, 400, 600, 800, 1000, 1200 μmol/(m^2^s)^-1^]. The CO_2_ concentration was 400 μmol·mol^-1^, the temperature was controlled at 27°C, the maximum photosynthetically active photon flow density was 300 μmol·s^-1^, the retardation rate was 30%, the modulation frequency was 20 kHz, and the averaging filter frequency was 50 Hz.

### 2.4 Community survey and biomass measure

Community surveys were conducted in August 2019, during the peak plant growth period. A 1m×1m standard sample plot was placed directly in the middle of each plot and all species in the sample plot were surveyed. Three plants of each species were randomly selected and their natural upright height was measured. Vegetation cover was determined by visual estimation. After the survey, the aboveground parts of all species in the sample square were harvested for both species subsequently, divided into species bagged, and brought back to the laboratory where they were placed in a 75°C oven for 48h and weighed to obtain the aboveground biomass (AGB) of the community. In the following text, the AGB means total above-ground biomass of the community, while the biomass of *B.ischaemum* and *L.davurica* is expressed by “biomass of *B.ischaemum* and *L.davurica*”.

The transgressive overyielding is a phenomenon in which the production of a mixture community is higher than the sum of the monoculture yield of each species within that mixture (Van der Werf et al., 2021). This is quantified using the relative yield total (RYT), which represents the ratio of the actual production of a species in the mixture community to its predicted production in its monoculture (Nyfeler et al., 2009). Specifically: If RYT is greater than 1, it indicates that there is an overyielding effect; if RYT is equal to 1, it indicates no overyielding effect; if RYT is less than 1, it means that there is a yield reduction effect.

The RYT is defined as:


$$RY_{B}=\frac{MB_{actual}}{MB_{predict}}=\;\;MB_{mix}/\left(i\times \;\frac{MB_{single}}{10}\right)$$

$$RY_{L}=\frac{ML_{actual}}{ML_{predict}}=\;\;ML_{mix}/\left(j\times \;\frac{ML_{single}}{10}\right)$$

$$\text{RYT}=\;RY_{B}+RY_{L}$$

Here, RY_B_ is the relative production of *B.ischaemum* in the mixture community, MB_actual_ is the actual production in the mixture community, MB_predict_ is the predicted production in the monoculture, and *i* is the mixture ratio of *B.ischaemum.* Similarly, RY_L_ is the relative production of *L.davurica* in the mixture community, MB_actual_ is the actual production in the mixture community, MB_predict_ is the predicted production in the monoculture, and *j* is the mixture ratio of *L.davurica.*

### 2.5 Determination of plant and soil nitrogen and phosphorus content

After drying and weighing, the aboveground parts of the plants were ground by a pulverize. After the community survey, three soil samples were randomly collected at 0–30 cm depth using a 2 cm soil auger in each plot. Soil samples were divided into two parts, one was used for soil moisture content (SMC) measurement, and the other part was allowed to air dry naturally. Air-dried soils were ground through a 0.25 mm sieve to determine soil total nitrogen (STN), and soil total phosphorus (STP) contents. To differentiate from the soil nutrient content in 2019, background values for 2018 were expressed using STN_2018_ and STP_2018._ The nitrogen content of *B.ischaemum* (N_Bi_), *L.davurica* (N_Ld_), and soil total nitrogen (STN) was determined by Kjeldahl nitrogen determination. The phosphorus content of *B.ischaemum* (P_Bi_), *L.davurica* (P_Ld_), and soil total nitrogen (STP) was determined by the molybdenum blue colorimetric method.

### 2.6 Data and statistical analyses

Before the analysis of variance (ANOVA), the Shapiro–Wilk test was used to assess normality and an F-test to assess the homogeneity of variance. A one-way analysis of variance (ANOVA) was used to compare the significant differences in fluorescence parameters (Fo, Fv/Fm, ΦPSII, NPQ, qP) and plant nutrient content (nitrogen and phosphorus) between *B.ischaemum* and *L.davurica* under the same treatment (same mixture and same N level) (*p*< 0.05). Next, two-way ANOVA was used to analyze the significant differences between the treatments (mixture and N addition) in fluorescence parameters (Fo, Fv/Fm, ΦPSII, NPQ, qP), plant nutrient content (nitrogen and phosphorus), soil nutrient content (nitrogen and phosphorus), aboveground biomass, and overyielding (RY_B_, RY_L_, RYT) (*p*< 0.05). Furthermore, to examine the effects of the pre-test soil characteristics, the soil total nitrogen (STN_2018_) and soil total phosphorus (STP_2018_) in 2018 were included as covariates in the two-way ANOVA. Since they were not measured at the same time, we did not consider the interaction of the covariates with mixture and nitrogen addition. All statistical analyses were performed with SPSS 22.0 (SPSS Inc., Chicago, IL, USA). After ANOVA, the Turkey method was used for multiple comparisons of between-group differences.

The partial least squares path models (PLS-PM) provide a framework to test multivariate hypotheses that the complex relationship among mixture ratio, N addition, soil nutrients, plant nutrients, chlorophyll fluorescence, and community production (Hu et al., 2021). According to previous studies, exogenous N addition and mixture ratio both have direct effects on soil nutrients, and plant nutrients (Schipanski and Drinkwater, 2012; Chen et al., 2020b; Li et al., 2015; Xu et al., 2016). Furthermore, plant nutrients affect community AGB by directly influencing species’ fluorescence parameters (Hallik et al., 2012; Yue et al., 2023). Moreover, the soil background nutrients might have effects on the community (Janušauskaite and Feiziene, 2012). The mixture ratio was characterized as an exogenous latent variable, reflected by two observed variables, that was the mixture ratio of *B.ischaemum* and *L.davurica*. The ‘soil conditions’ is an endogenous latent variable, characterized by soil moisture content, soil total nitrogen, and total phosphorus; The ‘plant nutrient’ is also an endogenous latent variable, which is directly affected by the plant N content, P content, N:P ratio of *B.ischaemum* and *L.davurica*, respective. Among the fluorescence parameters, the pathway coefficients of Fv/Fm, ΦPSII of *B.ischaemum* were not taken into account because of its too low effect affecting the overall fitting accuracy of the model. Similarly, the Fv/Fm and NPQ of *L.davurica* were not considered in the path. Correlation significant test at *p*< 0.05 level. Evaluate the overall predictive performance of the model using goodness-of-fit metrics, and the PLS- PM analyses were done with the ‘plspm’ package in R 4.3.2.

## 3 Results

### 3.1 Response of chlorophyll fluorescence parameters to N addition in *B.ischaemum* and *L.davurica* mixture community

**Figure 2:**
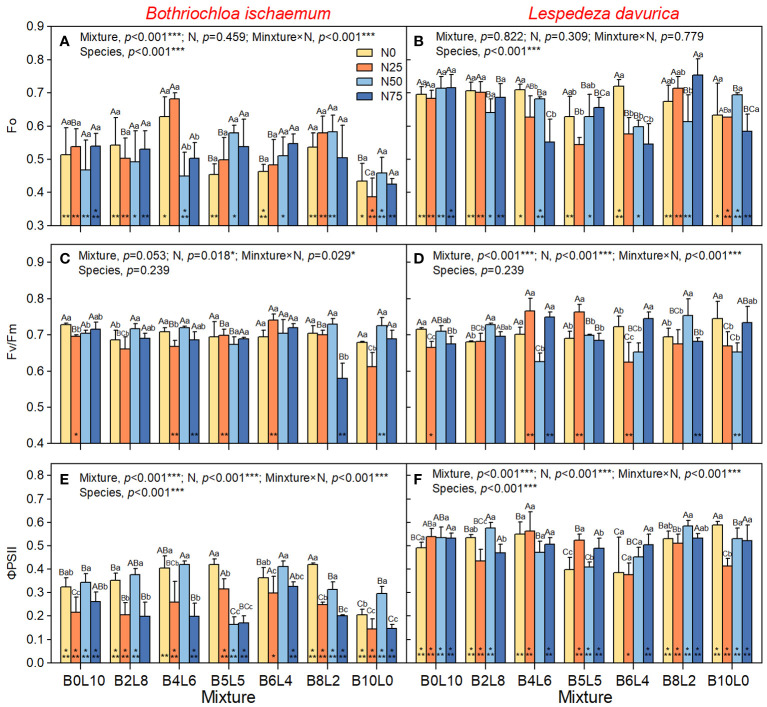
Response of chlorophyll fluorescence of Bothriochloa ischaemum and Lespedeza davurica to mixture ratios and nitrogen additions. **(A)** Fo of B.ischaemum; **(B)** Fo of L.davurica; **(C)** Fv/Fm of B.ischaemum; **(D)** Fv/Fm of L.davurica; **(E)** FPSII of B.ischaemum; **(F)** FPSII of L.davurica. The asterisk * indicate significant differences between B.ischaemum and L.davurica in same treatment at p< 0.05. Different capital letters indicate significant differences among the mixture ratio in the same N addition levels at p< 0.05; Different lowercase letters indicate significant differences among the N addition levels in the same mixture ratio at p< 0.05.

**Figure 3:**
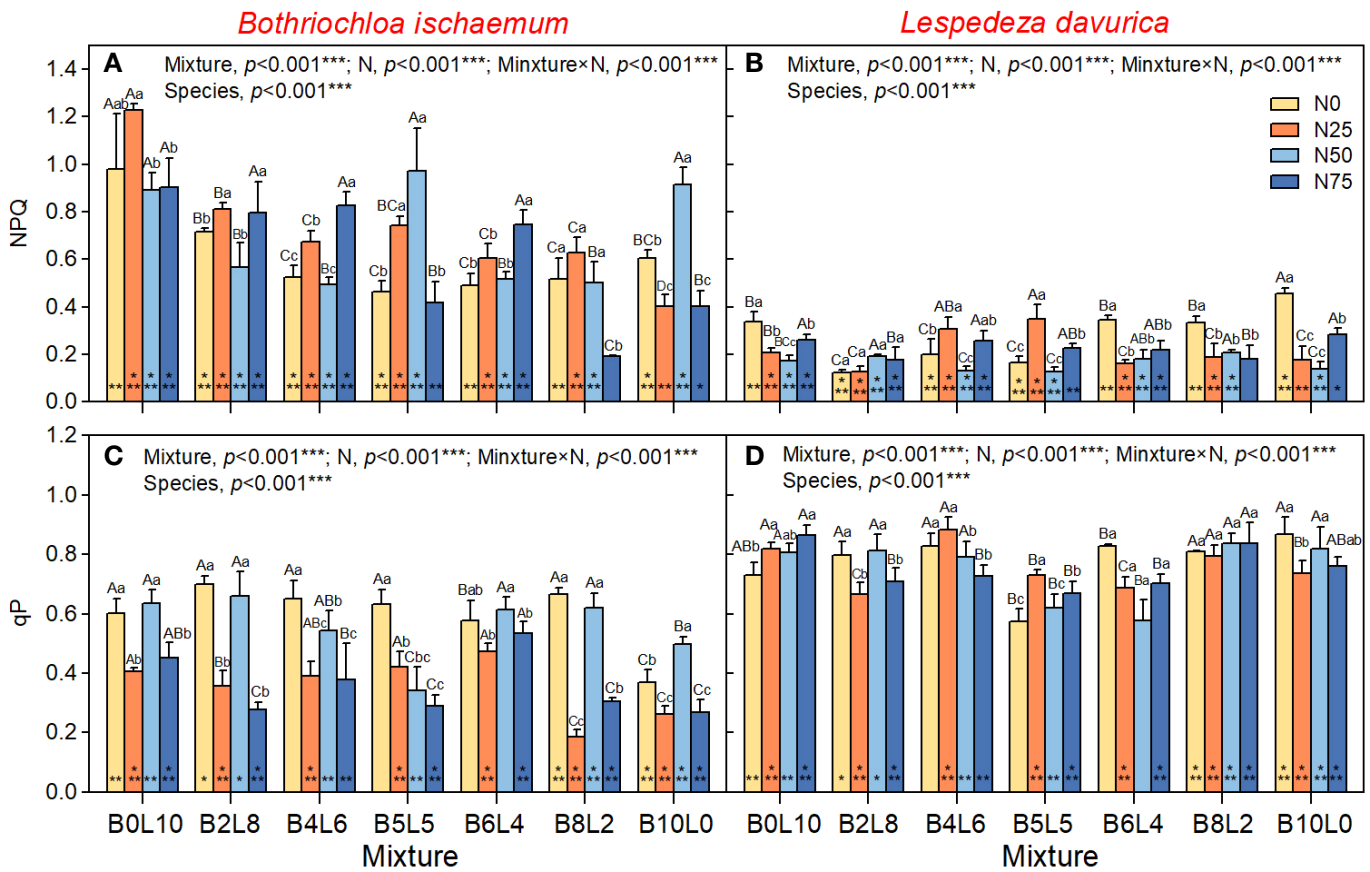
Response of non-photochem-ical fluorescence quenching (NPQ), coefficient of photochemical fluorescence quenching (qP) of B.ischaemum and L.davurica to mixture ratios and nitrogen additions. **(A)** NPQ of B.ischaemum; **(B)** NPQ of L.davurica; **(C)** qP of B.ischaemum; **(D)** qP of L.davurica. The asterisk * indicate significant differences between B.ischaemum and L.davurica in same treatment at p< 0.05. Different capital letters indicate significant differences among the mixture ratio in the same N addition levels at p< 0.05;Different lowercase letters indicate significant differences among the N addition levels in the same mixture ratio at p< 0.05.

One-way ANOVA results showed that there was a significant difference in Fo, PSII, NPQ, and qP between *B.ischaemum* and *L.davurica* in the same community (*p*<0.05). The Fo, ΦPSII, and qP of *L.davurica* were significantly higher than those of *B.ischaemum*, but there was no significant difference in FV/Fm, while the NPQ of *B.ischaemum* was significantly higher than that of *L.davurica* (**Figures 2**, **3**).

The results of the two-way analysis (ANOVA) showed that STN_2018_ had significant effects on Fo of *B.ischaemum*, and ΦPSII of *L.davurica*; STP_2018_ had significant effects on Fo of *B.ischaemum*, and NPQ of *L.davurica* (**Supplementary Table S1**). However, these effects were relatively smaller than mixture alone, N addition alone, and mixture × N interaction, which all have significant effects on Fv/Fm, ΦPSII, NPQ, and qP of *B.ischaemum* (**Figure 2**, *p*<0.05). The Fo and ΦPSII of *B.ischaemum* were significantly higher in the mixture communities than in its monoculture under all N treatments (**Figures 2A, C**, *p*<0.05). N addition significantly increased Fo of *B.ischaemum* in the mixture communities but decreased the ΦPSII (**Figure 2E**). NPQ significantly decreased with the mixture ratio from 0:10 to 10:0 (grass-legume), while there was no significant difference in Fv/Fm and qP. Under N50 treatment, the PSII and qP of *B.ischaemum* were significantly higher than those of N20 and N75, without relation to the mixture ratio. In the monoculture community of *B.ischaemum*, the N25 treatment significantly reduced the Fo, FV, ΦPSII, NPQ, and qP, but the N50 treatment significantly increased fluorescence.

For *L.davurica*, the mixture ratio and N interaction all have a significant impact on Fv/Fm, ΦPSII, NPQ, and qP, except for Fo. Specifically, the Fv/Fm, ΦPSII, and qP were significantly lower under B5L5 and B6L4 treatment than those under monoculture (B0L10). Under the monoculture of *L.davurica*, N addition did not affect Fo and ΦPSII but significantly reduced the Fv/Fm and NPQ (**Figures 2B, D, F**). Under the mixture community, the Fo and qP gradually decreased with the grass-legume ratio and was significantly lowest at B5L5. Whereas there were no significant differences in Fv/Fm, ΦPSII NPQ. Under N0 treatment, there was no significant difference in fluorescence of *L.davurica* between monoculture and mixture (except for B5L5, which was significantly the lowest). The effect of N addition was affected by the mixture ratio. Under B4L6 and B5L5, N25 treatment significantly increased the Fv/Fm ΦPSII, NPQ, and qP of *L.davurica*.

### 3.2 Response of the rapid light-response curves of chlorophyll fluorescence

**Figure 4:**
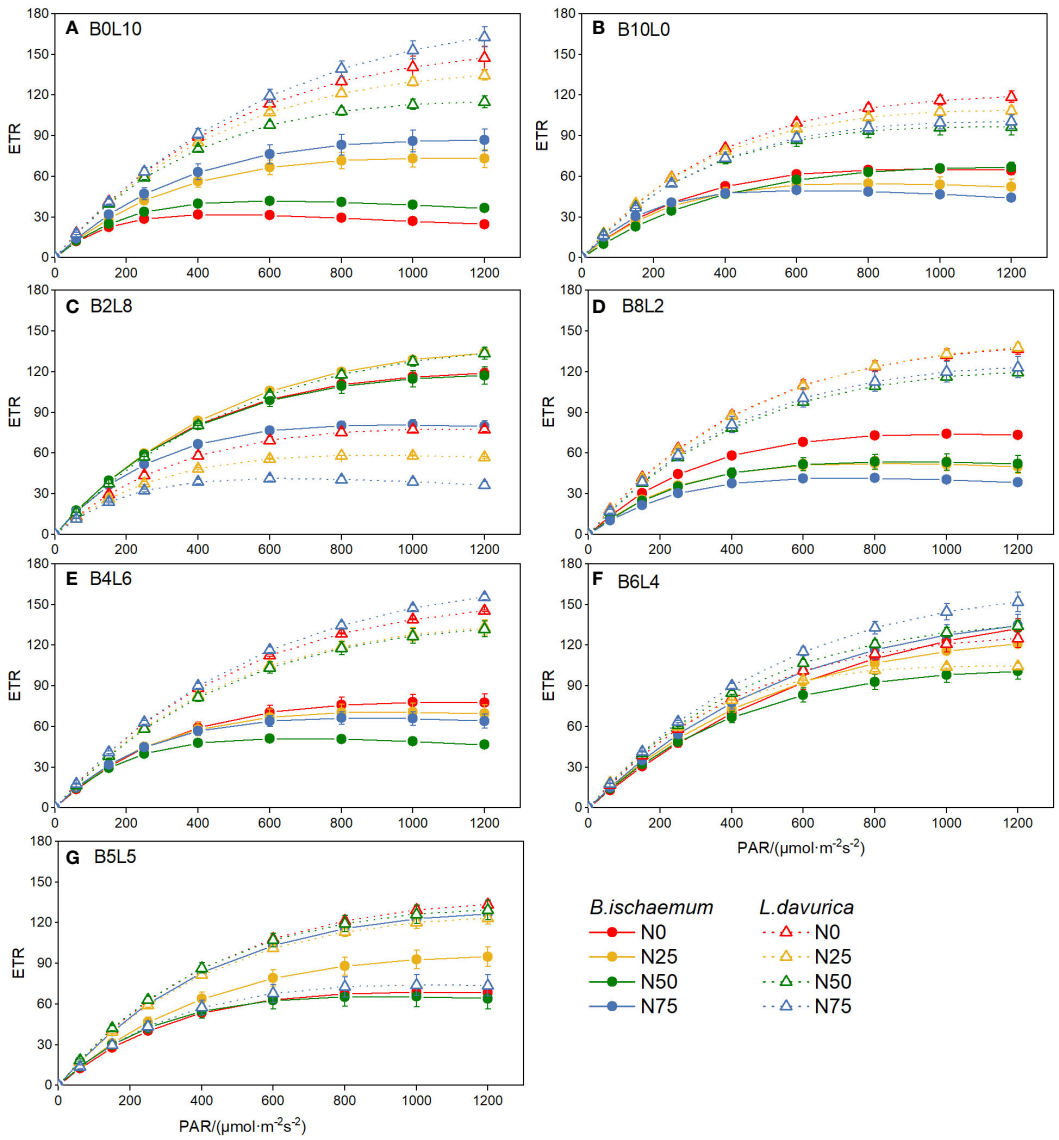
The rapid light-response curves of chlorophyll fluorescence of B.ischaemum and L.davurica in different mixture ratios and nitrogen levels. **(A)** B0L10; **(B)** B10L0; **(C)** B2L8; **(D)** B8L2; **(E)** B4L6; **(F)** B6L4; **(G)** B5L5.

The rapid light-response curves of *B.ischaemum* and *L.davurica* showed significant differences under different mixture ratios and N addition treatments, indicating various light utilization and dissipation strategies (**Figure 4**). In the two monoculture communities (B0L10 and B10L0), the changing rate of ETR with PAR of *L.davurica* was significantly higher than that of *B.ischaemum*.

In mixture communities (except for B2L8 and B4L6), the changing rate of ETR with PAR of *L.davurica* was also higher than that of *B.ischaemum*, indicating that *L.davurica* had stronger adaptability to high light intensity, while *B. ischaemum* was relatively weaker. Compared with monoculture (B10L0 and B0L10), the mixture decreased *L.davurica’s* ETR. In mixture treatments, there was no significant difference in the ETR of *L.davurica* among different N levels. The ETR of *L.davurica* under N0 was higher than that of N25 and N50, and N addition had an inhibitory effect on the photosynthesis of *L.davurica*. The ETR of *B.ischaemum* increased first and then decreased as the mixture ratio of *B.ischaemum* increased. It was significantly highest in B6L4 and lowest in B10L0.

### 3.3 Effect of N addition on the biomass in *B.ischaemum* and *L.davurica* mixture community

**Figure 5:**
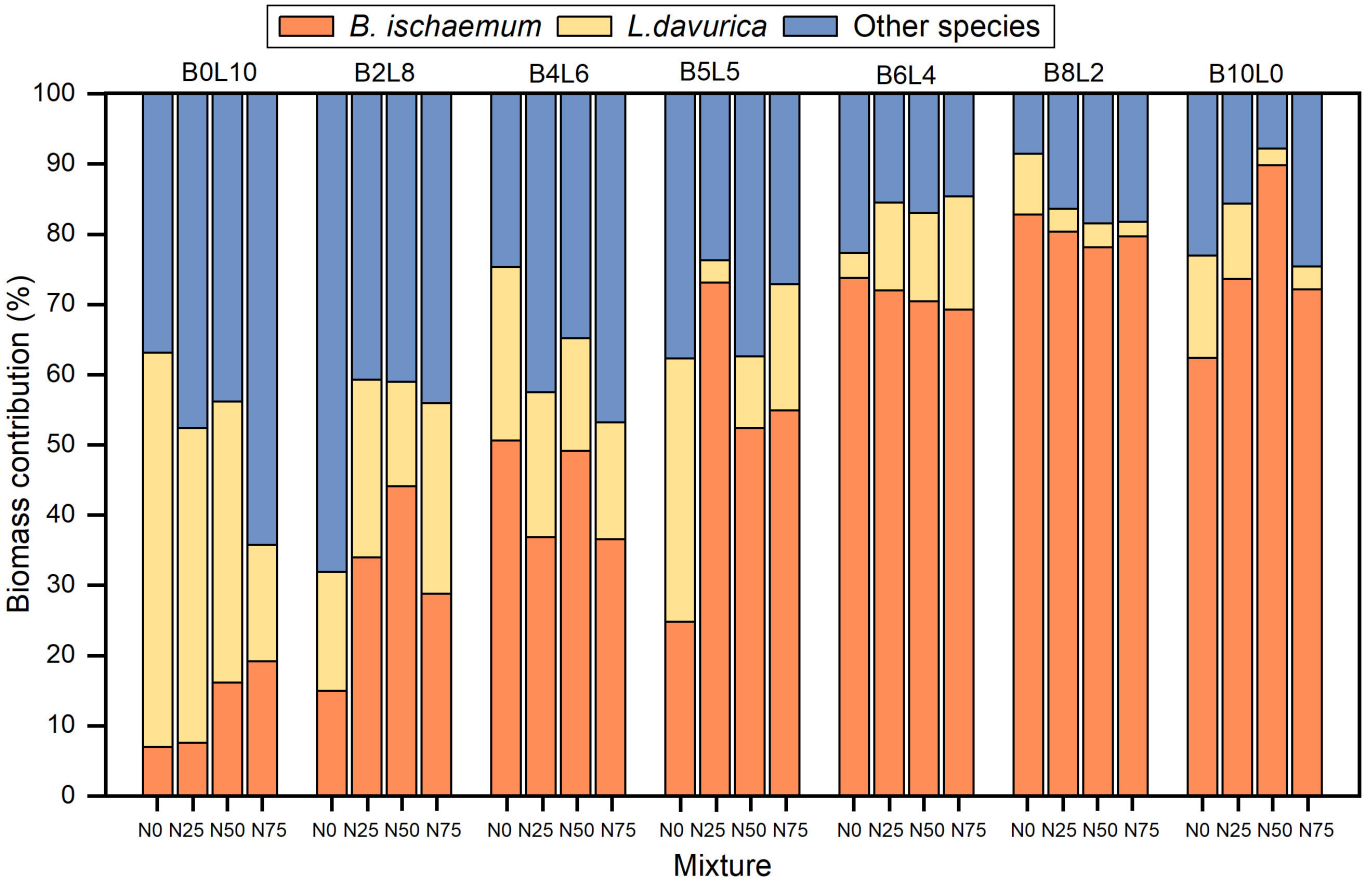
The biomass contribution difference of *B.ischaemum*, *L.davurica* and other species in different mixture and N addition communities.

The biomass contribution of *B.ischaemum* and *L.davurica* was highly correlated with their mixture ratio, but N addition changed the biomass contribution of species (**Figure 5**). The proportion of biomass of other species in the *L.davurica* monoculture community (B0L10) was significantly higher than that of *B.ischaemum* (B10L0). As the N addition levels increased, the biomass contribution of *B.ischaemum* and other species both increased, at the expense of a decrease in *L.davurica* in the *L.davurica* monoculture community. The biomass contribution of *B.ischaemum* was significantly higher than that of other species in *B.ischaemum* monoculture and B6L4, B8L2 mixture communities, occupying a dominant ecological niche. As the mixture ratio of *B.ischaemum* increased, the biomass contribution of *L.davurica* and other species significantly decreased. The biomass contribution of *L.davurica* in B6L4 significantly increased with N addition levels.

**Figure 6:**
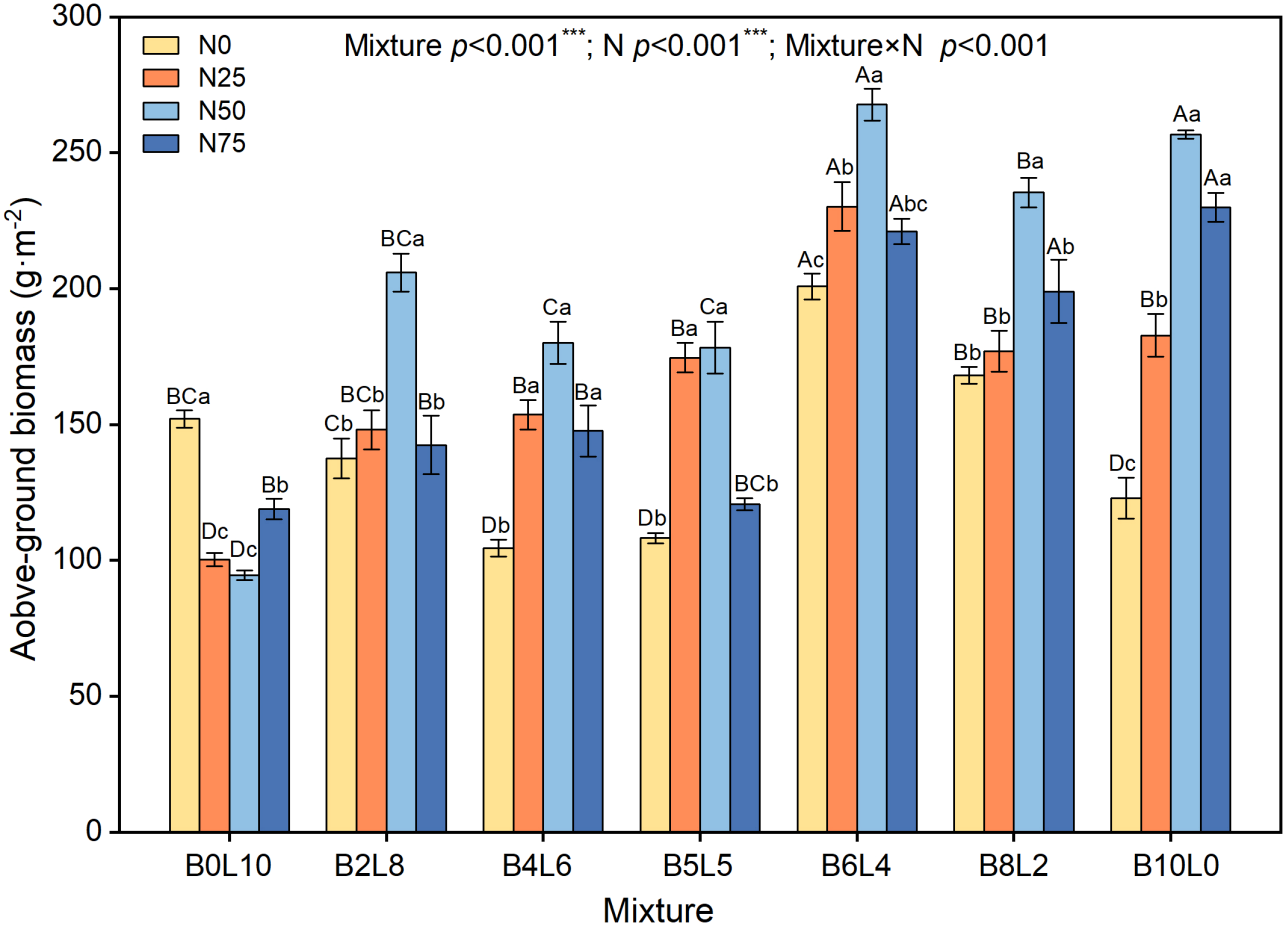
Response of community aboveground biomass to mixture ratios and nitrogen additions. Different capital letters indicate significant differences among the mixture ratio in the same N addition levels at *p*< 0.05; Different lowercase letters indicate significant differences among the N addition levels in the same mixture ratio at *p*< 0.05. ***means P ≤ 0.001, ** means P ≤ 0.01, *means P ≤ 0.05.

The AGB in the *B.ischaemum* monoculture was significantly higher than that of the *L.davurica* monoculture (**Figure 6**). Under the same N level, the AGB of the mixture community was significantly higher than that of the monoculture of *L.davurica* (B0L10). Compared with N0, the AGB increased and then decreased with N addition levels. Under N0, AGB showed a significant decrease from B0L10 to B5L5, with B6L4 being significantly the highest, then B8L2 and B10L0 showed a linear decrease. The AGB under N50 was significantly higher than that of N0, N25, and N75 in both monoculture (B10L0 and B0L10) and mixture communities. Under N25 treatment, AGB first increased and then decreased with mixture ratio, being significantly highest in B6L4. At the same N level, the AGB in B6L4, B8L2, and B10L0 treatments were significantly higher than that of B0L10, B2L8, B4L6, and B5L5. These results showed that the higher the mixture ratio of *B.ischaemum*, the greater the biomass contribution.

### 3.4 Effect of N addition on the overyielding in *B.ischaemum* and *L.davurica* mixture community

**Figure 7:**
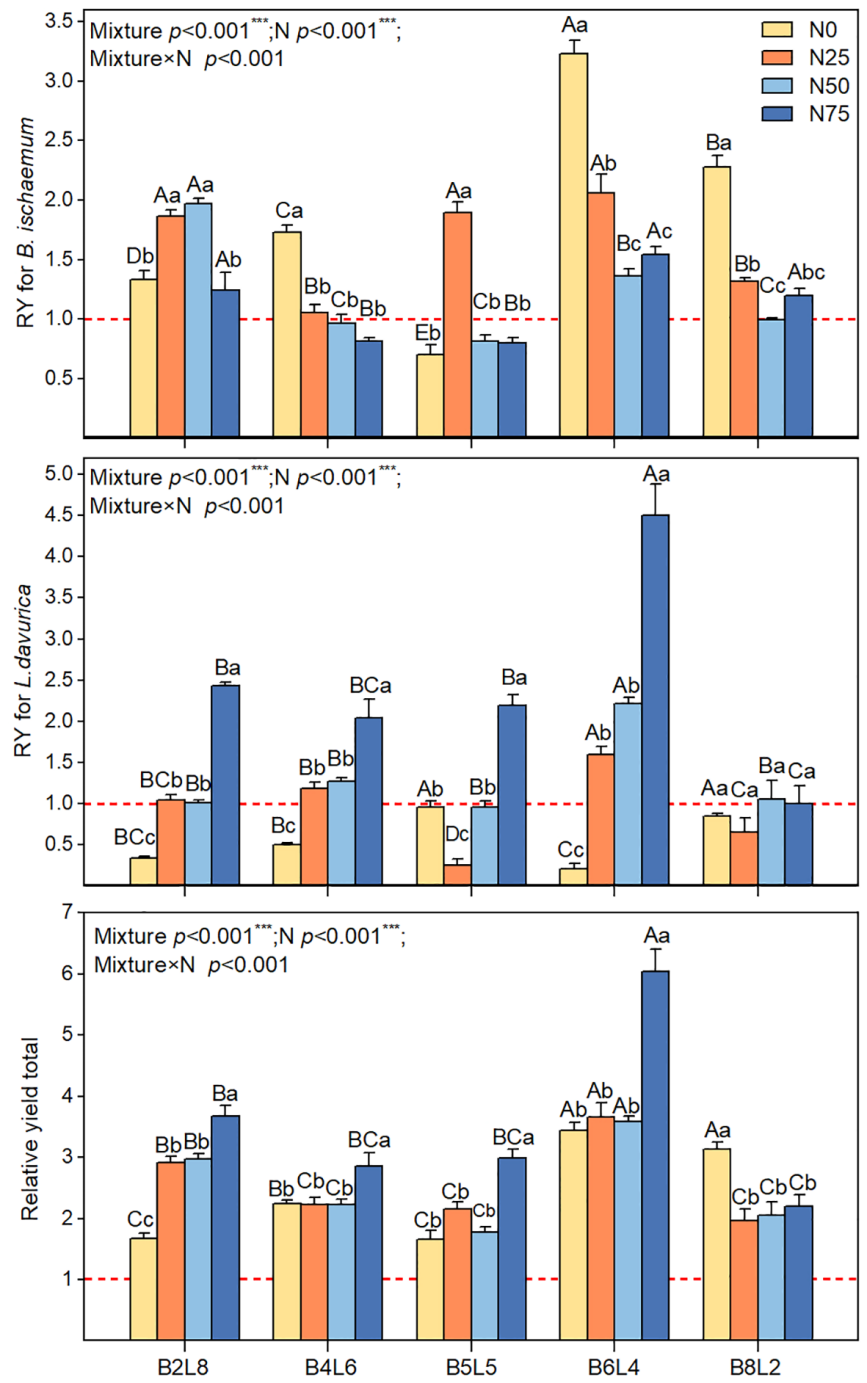
The relative yield (RY) for *B.ischaemum*, for *L.davurica*, and relative yield total (RYT) for community in different N addition and mixture ratio treatments. Different capital letters indicate significant differences among the mixture ratio in the same N addition levels at *p*< 0.05; Different lowercase letters indicate significant differences among the N addition levels in the same mixture ratio at *p*< 0.05. ***means P ≤ 0.001, ** means P ≤ 0.01, *means P ≤ 0.05.

**Figure 7** showed that the RYT was greater than 1 under all mixture communities, indicating a significant overyielding effect. However, there were significant differences between different mixture ratios or N treatments. Under N0, the overyielding effects of mixture communities were attributed to *B.ischaemum*’s overyielding, whereas *L.davurica* produced yield reduction effects. The total relative yield (RYT) of mixture communities was significantly higher under N75 than that of N0, N25, and N50 treatments. N75 significantly improved the relative yield of *L.davurica* in mixture communities.

### 3.5 PLS-PM detects the relationship among mixture ratio, N addition, soil and plant nutrients, chlorophyll fluorescence parameters, and AGB

**Figure 8:**
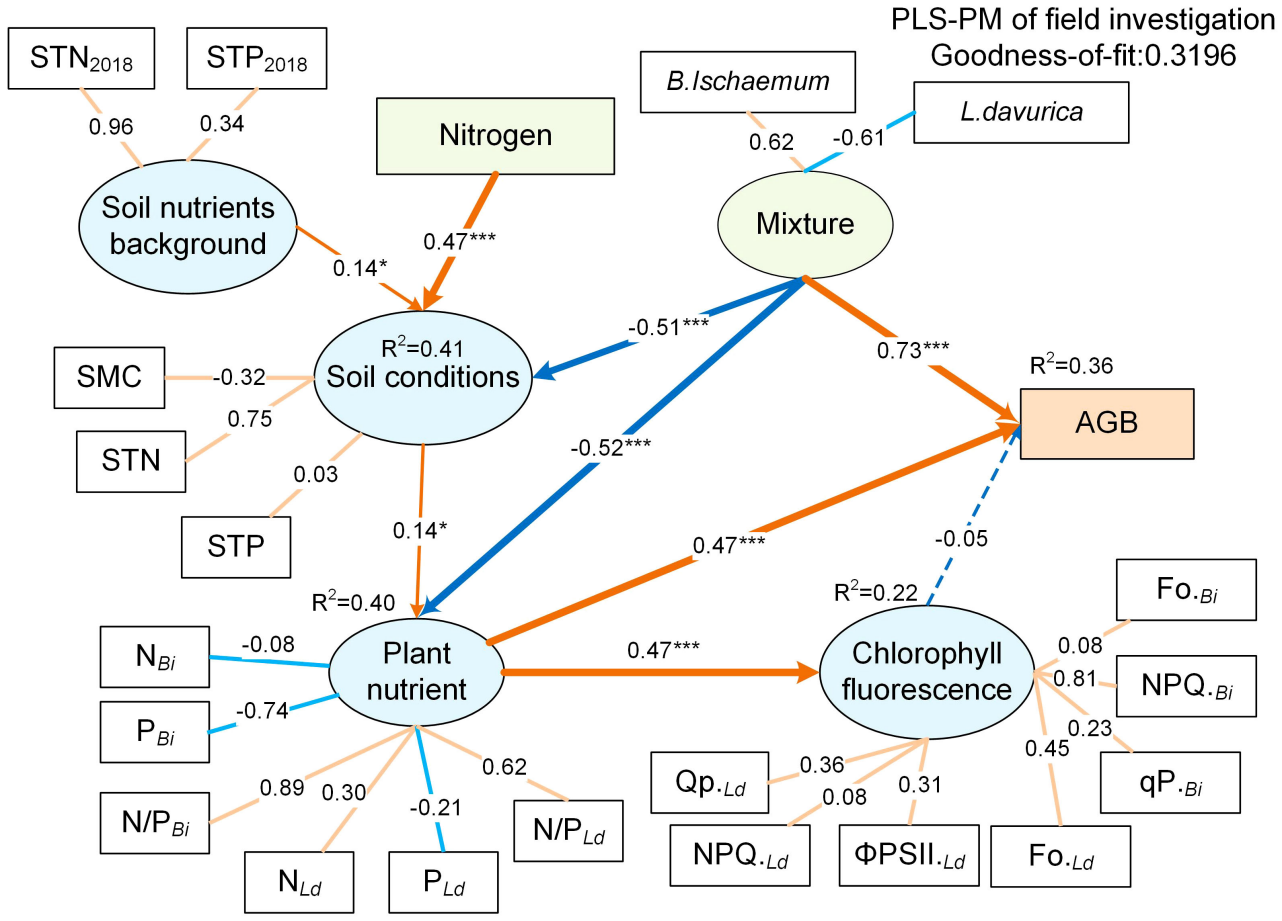
Partial least squares path models (PLS-PM) for testing the effects of mixture, N addition on soil nutrient, plant nutrient, plant fluorescence and AGB. The gradual arrow widths are proportional to the strength of the path coefficient. Orange lines indicate positive impacts (*p*< 0.05), blue lines indicate negative impacts (*p*< 0.05), and dashed lines indicate path coefficients that are not significant (*p* > 0.05). Correlation significant test at *p*< 0.05 level. Reflective latent variables (ellipse) are indicated by measured variables with their respective loadings shown. ***means P ≤ 0.001, ** means P ≤ 0.01, *means P ≤ 0.05.

The PLS-PM analysis revealed the relationships between mixture ratio, N addition, and community biomass (**Figure 8**). When the STN_2018_ and STP_2018_ (covariate) were taken into account in statistical models, soil nutrients before fertilization only had a weak negative effect on SMC, STN, and STP (r=0.14). N addition had a significant direct positive effect on soil nutrients (0.47, *p*<0.001) but had a weak indirect effect on plant nutrients, fluorescence, and AGB (0.07, 0.03, -0.03) (**Supplementary Figure S2**). Mixture ratio had a direct negative effect on soil nutrients and plant nutrients (-0.51, -0.56, *p*<0.001), and a significant direct positive effect on AGB (0.73). The direct effects of fluorescence parameters on AGB were very slight (r=-0.05, p>0.05), whereas the direct effects of plant nutrients on AGB were stronger (r=0.47, *p*<0.001). The biomass contribution of *B.ischaemum* was positively correlated with the mixture, while the biomass contribution of *L.davurica* was negatively correlated, indicating that the higher the mixture ratio of *B.ischaemum*, the higher the community AGB. This was consistent with the results of the analysis of variance (**Figure 6**). Interestingly, plant nutrients had the same positive effect on both fluorescence parameters and AGB (0.47, *p*<0.001). The correlation between STN and soil nutrients reached 0.86, indicating that soil nutrients were mainly affected by soil N. Among plant nutrients, the N_Bi_ was significantly negatively correlated with P_Bi_, and the N_Ld_, and P_Ld_, indicating that the nitrogen utilization strategy of *B.ischaemum* was different from that of *L.davurica*.

## 4 Discussion

### 4.1 Effects of N addition on the chlorophyll fluorescence in *B.ischaemum* and *L.davurica* mixture community

The chlorophyll fluorescence and rapid light-response curves represent plant photosystem processes such as light energy absorption, transmission, dissipation, and distribution, which play significant roles in plant biomass accumulation (Zhao et al., 2019). Chlorophyll fluorescence parameters can be easily measured and provide useful probes of photosynthetic performance *in vivo* and the extent to which performance is limited by photochemical and nonphotochemical processes (Baker, 2008). In this study, *L.davurica* had significantly higher maximum fluorescence (Fo), photochemical efficiency (ΦPSII), and photochemical quenching coefficient (qP) than *B.ischaemum*, indicating a more active photochemical reaction and efficient utilization of absorbed light energy. Similarly, the rapid light-response curves showed a consistent pattern, the ETR of *L.davurica* increased with PAR higher than that of *B.ischaemum*, indicating that *L.davurica* had stronger adaptation to high photosynthetically active radiation, and higher photosynthetic capacity and production (Liu et al., 2016). On the contrary, the non-photochemical quenching (NPQ) of *B.ischaemum* was higher than that of *L.davurica*, suggesting that *B.ischaemum* was able to dissipate excitation energy as heat more strongly, thus protecting photosynthesis from photoinhibition (Baker, 2008). Furthermore, the significant differences in rapid light-response curves of *B.ischaemum* and *L.davurica* indicate various light utilization and dissipation strategies of these two species. In this study, the changing rate of ETR with PAR of *B. ischaemum* increased and then decreased, showing a clear photoinhibition phenomenon. This phenomenon could be attributed to the different upper photochemical and non-photochemical pathways in the membranes of C3, and C4 plant-like vesicles, as well as the ecological niches of the two plants (Lin et al., 2013; Guidi et al., 2019). Studies have revealed that C4 species are more adaptable to temperature, photosynthetically active radiation, and carbon dioxide concentration, allowing for efficient photosynthesis in high temperature, high light, and low carbon dioxide environments. In contrast, photosynthesis in C3 plants is subject to suppression by high temperature, high light, and low carbon dioxide (Baker, 2008; Guidi et al., 2019). In summary, *L.davurica* and *B.ischaemum* exhibited different photosynthetic acclimatization strategies, with *L.davurica* having higher photosynthetic capacity and photosynthetic yield, whereas *B.ischaemum* having stronger photoprotective capacity and photosynthetic stability.

The differences in the photosynthetic strategies of the two species in the above results are not only because of the genetic differences between the two species, the interaction of mixture and N addition greatly also affected the photosynthetic strategies and adaptive mechanisms (Liu et al., 2016). Mixture ratio affected the photosynthetic strategies mainly by altering interspecific relationships within the community, while N addition through nutrient cycling (Nyfeler et al., 2009; Schleuss et al., 2020; Abalos et al., 2021). In this study, the rate of electron transport rate (ETR) change with photosynthetically active radiation (PAR) was significantly higher in the monoculture of *L. davurica* (B0L10) compared to that in the mixture community. Additionally, non-photochemical fluorescence quenching (NPQ) decreased with increasing mixture ratio, suggesting that the mixture enhanced light dissipation in *L.davurica* and consequently reduced its photosynthetic efficiency. The Fv/Fm and ΦPSII of *L.davurica* decreased and then increased with mixture ratio, and showed an inflection point at B5L5 treatment, indicating that the photosynthetic efficiency of *L.davuric* was affected by the ratio of grasses. When *L.davuric* remains dominant in a mixture community, it can maintain a high photosynthetic capacity. However, as a companion species, its photosynthesis becomes constrained due to reduced light availability (Tejera et al., 2016). This limitation may be associated with *L.davurica*’s high photosynthetic production but relatively low light protection capacity. Given *L.davurica*’s shorter stature and creeping growth habit, the shading effect from the taller *B.ischaemum* canopy gradually intensifies, resulting in a competitive disadvantage for L. davurica in terms of light competition (Chen et al., 2020b). All fluorescence parameters of *B.ischaemum* in the mixture communities were significantly higher than those in the monoculture (**Figures 2**, **3**). When mixed with legumes, the gramineous species was able to benefit from N fixation by legumes, and N obtained in this way was much more than direct exogenous N addition (Schipanski and Drinkwater, 2012). The effect of the mixture on *B.ischaemum*’s ETR was higher than that of N addition and was highest in B6L4 treatment, which was not significantly different from that of *L.davurica*. The above results indicated that the photosynthetic efficiency of *B.ischaemum* reached its maximum in this specific mixture ratio, aligning with the observed pattern of ΦPSII (Xu et al., 2011). The divergent response patterns in chlorophyll fluorescence parameters between the two species across different mixture ratios reflect the dynamic interplay of coexistence and competition (Brophy et al., 2017). In summary, the mixture altered interspecific relationships, affecting light dissipation and photosynthetic efficiency.

Fertilization was the limit on photosynthetic efficiency, as evidenced by the decreasing effect of N addition on Fv/Fm and ΦPSII of *L.davurica* with N levels (Chen et al., 2020a). Conversely, as autotrophic nitrogen-fixing plants, high nitrogen input reduced the nitrogen and phosphorus content in the plant (**Supplementary Figures S2B, D**) (Xu et al., 2016). This reduction affects *L.davurica*’s photosynthetic carbon assimilation capacity and pigment synthesis, resulting in decreased chlorophyll content and photosynthetic rate (Wang et al., 2019). In contrast, the Fv/Fm and ΦPSII of *B.ischaemum* exhibit an inflection point in response to N levels, indicating a non-linear relationship. The weak effect of STN_2018_ and STP_2018_ on fluorescence parameters as well as soil nutrients after fertilization confirmed that exogenous N additions had a much stronger effect on the species than mixing ratios (**Figure 8** and **Supplementary Table S1**). Moderate N input promotes the photosynthetic efficiency of *B.ischaemum*, while excessive N inhibits photosynthesis. In this study, ΦPSII and qP of *B.ischaemum* under N75 treatment were significantly lower than in N25, and N50 in mixture communities, which had already adversely affected the photosynthesis, consistent with the findings of (Zhao et al., 2019). Studies of the brassica juncea found the same pattern, with N supply increasing light energy conversion efficiency and potential photosynthetic reaction center activity, but excessive N failing to promote their growth (Ullah et al., 2020). In summary, *B.ischaemum* and *L.davurica* revealed varied photosynthetic physiological responses under different mixtures and N addition treatments. Mixture increased ΦPSII of *B.ischaemum* but decreased photosynthesis in *L.davurica*. N addition can improve soil fertility, but it can also cause soil acidification, thus it is vital to adequately limit the amount and frequency of N addition to avoid its detrimental influence on grassland ecosystems (Wang et al., 2022). In summary, the photosynthetic responses of *B.ischaemum* and *L.davurica* varied under different mixture ratios and nitrogen (N) addition treatments. N addition adversely affected *L.davurica*’s photosynthesis, likely due to reduced nitrogen and phosphorus content.

### 4.2 Effects of mixture ratio and N addition on community above-ground biomass and overyielding

Consistent with the expected results, the mixture ratio significantly increased AGB and stimulated the overyielding effect (**Figures 6**, **7**) (Finn et al., 2013; Li et al., 2014; Liu et al., 2016). Without N addition, the community AGB in B2L8, B4L6, and B5L5 was significantly lower than that of *L.davurica* monoculture, suggesting that *L.davurica* contributed less to community AGB than *B.ischaemum* and other species. The biomass contribution of other species in B0L10, B2L8, and B4L6 was high (>50%) (**Figure 4**), implying that species invasion limited *L.davurica*’s growth and *L.davurica* could not maintain its dominance in the community indefinitely (Finn et al., 2013). It might be because legume species have shallow roots, low water use efficiency, short size, and relatively low resistance and adaptability (Tognetti et al., 2021). The overyielding in the B0L10, B2L8, and B4L6 treatments was mainly from *B.ischaemum*. The biomass contribution of *B.ischaemum* in the mixture communities was an important factor for the direct positive effect on overyielding (**Figure 8** and **Supplementary Figure S3**). This effect was associated with several factors, including competitive advantages in light resource utilization, efficient nitrogen uptake, and enhanced photosynthetic stability (higher photosynthetic yield) (Atwater and Callaway, 2015; Chen et al., 2020b). Previous pot experiment studies have indicated that the 8:4 ratio of *B.ischaemum* and *L.davurica* mixture exhibited significantly greater relative yield totals and enhanced biomass production (Xu et al., 2011). Additionally, both fertilization and the mixture proportions substantially enhanced plants’ N and P content by *B.ischaemum* (Xu et al., 2016). Although our results differed from those of the pot experiment, these results provided evidence that the optimal ratio of grass-legume mixture should not exceed 1:1. Species identity and functional composition influenced production and stability, the production of grasses was more stable across growing seasons (Küchenmeister et al., 2012). Although the abundance of *B.ischaemum* had a positive effect on community AGB, a higher ratio of *B.ischaemum* in the mixture did not result in increased production.

The community AGB after N addition in the B8L2 mixture remained smaller than that in B6L4, and *L.davurica* exhibited a significant yield reduction effect (**Figure 7**). Non-legumes benefit more from N additions by legumes, resulting in mixed mixtures surpassing non-legume monocultures but falling short of legume monocultures (Frankow-Lindberg and Dahlin, 2013). Our findings suggested that the *B.ischaemum* and *L.davurica* mixture should prioritize grass as the dominant species and legume as the companion species. Notably, the overyielding of *L.davurica* was significantly increased with N levels in B6L4 treatment, accompanied by substantial enhancements in community AGB and overyielding effects. Specifically, under N50 in the B6L4 treatment, both species exhibited higher ΦPSII values and reduced heat dissipation, indicative of optimal light energy conversion efficiency. A synergistic effect between *B.ischaemum* and *L.davurica*. was evident. Furthermore, Legumes can be used as an alternative measure to nitrogen fertilizer and legumes have the greatest ecological and economic potential. Li et al. (2015) concluded that the ratio of grasses to legumes is most reasonable when the ratio of grasses to legumes is 1:1. In summary, our study highlights the importance of considering both grass and legume species in mixed grassland communities. Specifically, the *B.ischaemum* and *L.davurica* mixture should prioritize grass as the dominant species and legume as the companion species. Under N50 in the B6L4 treatment, both species demonstrated optimal light energy conversion efficiency.

### 4.3 Relationship between chlorophyll fluorescence and production, and overyielding effects under mixture and N addition

The overyielding effect of the mixture community varied with the level of N addition, suggesting that the production depended not only on species composition and abundance but also the external environmental factors. The chlorophyll fluorescence reflects the photosynthetic capacity, allowing rapid detection of metabolic changes in a plant’s photochemical system under environmental stress or disturbances (Schreiber et al., 1995; Baker, 2008). In the absence of N addition, L. davurica’s nitrogen-fixing capacity may be sufficient to meet N limitations (Schipanski and Drinkwater, 2012), but other species exhibit greater competitiveness for effective soil N, resulting in yield reduction across mixture ratios. Remarkably, the relative yield total (RYT) was significantly highest under N75, suggesting that N application stimulated overyielding effects in mixed communities (Nyfeler et al., 2009). In general, the mixture increased the species diversity and functional diversity of the community, and improved community stability and resilience, while N addition decreased diversity (Rajaniemi, 2002; Tejera et al., 2016). The interaction between mixture and N addition affects the competitive and complementary relationships between the two species, which in turn affects the structure and function of the community.

The results of PLS-PMs indicated that N addition significantly increased soil total nitrogen and positively affected plant nutrients (**Supplementary Figure S1**). The effective nitrogen input promoted the production of plant photochemical reaction enzymes and chlorophyll, and increased plant photosynthesis (Guidi et al., 2019). Complementary utilization of nutrients by *B.ischaemum* and *L.davurica* in the community significantly increased the photosynthetic efficiency, but the effect of N on community AGB was counteracted due to the difference between the two species’ photosynthetic strategies (Cui et al., 2023). This discrepancy may arise because the ΦPSII and qP of *L.davurica* were higher than *B.ischaemum*, and the photosynthetic center passively received more light energy under the mixture and N addition combined influence, resulting in damage to the photosynthetic apparatus (White and Critchley, 1999). In contrast, *B.ischaemum* being more stable and adapted to high light conditions, exhibited higher non-photochemical quenching (NPQ), allowing for the dissipation of excess light energy as a self-defense mechanism (Hallik et al., 2012). Consequently, both N addition and mixture can promote *B.ischaemum* productivity.

The overyielding effects of *B.ischaemum* were higher than those of *L.davurica* across all mixture ratios, suggesting that *L.davurica*’s nitrogen fixation process was not efficiently converted to biomass. Grasses, on the other hand, exhibited higher efficiency in converting nitrogen uptake to biomass (van Paassen et al., 2020). The accumulation of biomass led to increased water consumption by plants, and water limitation may have hindered the expression of rhizobia activity (Schubert, 1995). Excessive nitrogen addition not only disrupted legume species but also resulted in interspecific asymmetric competition, leading to reduced community diversity and ultimately compromising grassland stability and resistance (Verma and Sagar, 2020). Another important result was that N addition did not increase community productivity indefinitely; the overyielding effects of *B.ischaemum* and *L.davurica* were not the highest under N50, but the AGB was significantly highest. The N50 treatment not only promoted synergistic effects of grass-legume, but also increased the biomass accumulation of other species, and the positive correlation between diversity and production was particularly essential (Lü et al., 2019). Furthermore, the plant nitrogen and phosphorus levels of *B.ischaemum* were negatively linked with those of *L.davurica*, indicating that their ecological niches for nutrient application were complementary (**Figure 7**; **Supplementary Figure S2**). In addition, the plant nitrogen and phosphorus contents of *B.ischaemum* were negatively correlated with those of *L.davurica*, indicating that their ecological niches for nutrient application were complementary (**Figure 7**; **Supplementary Figure S1**). The highest threshold for N addition in this study region was 50 kg N km^-2^ a^-1^, which was less than the thresholds for temperate grasslands (90 kg N km^-2^ a^-1^) and meadow grasslands (75 to 200 kg) (Tang et al., 2017) (Verma and Sagar, 2020). N application of 90 kg km^-1^ decreased light damage in the photosystem of Naked oat (*Avena nuda L.*), which in turn increased the yield during the grain-filling stage in naked oat monoculture grassland (Lin et al., 2013). Naked oats, which are classified as C3 gramineous plants, have higher carbon absorption efficiency than C4 plants but lower assimilate transport efficiency (Guidi et al., 2019), which was why its nitrogen need threshold was higher than that of *B.ischaemum*, another C3 plant in this study. In summary, the complex effects of N addition and mixture on soil nutrients, plant nutrients, and fluorescence parameters suggested that the soil-plant is a complex dynamic system that continually shapes the current community structure and function through the matter circulation and energy flow (Nyfeler et al., 2009). Taken together, it is necessary to choose reasonable mixture ratios and N addition levels to achieve functional optimization and balance in grassland ecosystems, which provides an important implication for assessing and predicting the stability and sustainability of grassland ecosystems.

## 5 Conclusion

This study revealed significant effects of nitrogen (N) addition on plant nutrients, chlorophyll fluorescence, and production in grass-legume mixture communities. The competitive and complementary relationship between *B.ischaemum* and *L.davurica* was evident. Notably, *L.davurica* exhibited higher photosynthetic capacity and yield, while *B.ischaemum* demonstrated stronger photoprotective capacity and photosynthetic stability. The N50 treatment enhanced the photosystem II efficiency of *B.ischaemum* when it dominated the mixture communities. However, the N addition negatively impacted the photosynthetic capacity of *L.davurica* in other mixtures.

The result of PLS-PMs highlighted that N addition increased soil nutrients, leading to improved photosynthetic efficiency through complementary nutrient utilization by both species. Although this positively influenced community productivity, the effect on aboveground biomass (AGB) was species-specific. The B6L4 ratio combined with N50 emerged as the optimal ratio treatment, maximizing photosynthetic electron transfer efficiency while minimizing heat dissipation. In summary, for grass-legume mixtures in the Loess Plateau, balancing species composition and N availability is crucial for sustainable productivity and ecosystem stability. This study can provide enlightenment for future construction of the grass-legume mixture artificial grassland in the Loess Plateau.

## Data availability statement

The original contributions presented in the study are included in the article/**Supplementary Material**. Further inquiries can be directed to the corresponding author.

## Author contributions

FW: Writing – original draft, Methodology, Investigation, Formal analysis, Conceptualization. LS: Writing – original draft, Visualization, Investigation, Formal analysis. RZ: Writing – review & editing, Formal analysis, Data curation. WX: Writing – review & editing, Supervision, Resources, Project administration, Funding acquisition, Conceptualization. YB: Writing – review & editing, Project administration.
