# Supplementary material for: Effects of nitrogen addition and Bothriochloa ischaemum and Lespedeza davurica mixture on plant chlorophyll fluorescence and community production in semi-arid grassland
Source: Front Plant Sci. 2024 Jun 24;15:1400309. doi: 10.3389/fpls.2024.1400309 (PMC11232416; doi:10.3389/fpls.2024.1400309)

**Fig S1** Response of soil total nutrient content, soil total phosphorus content, and soil N:P ratio to mixture ratios and nitrogen additions.


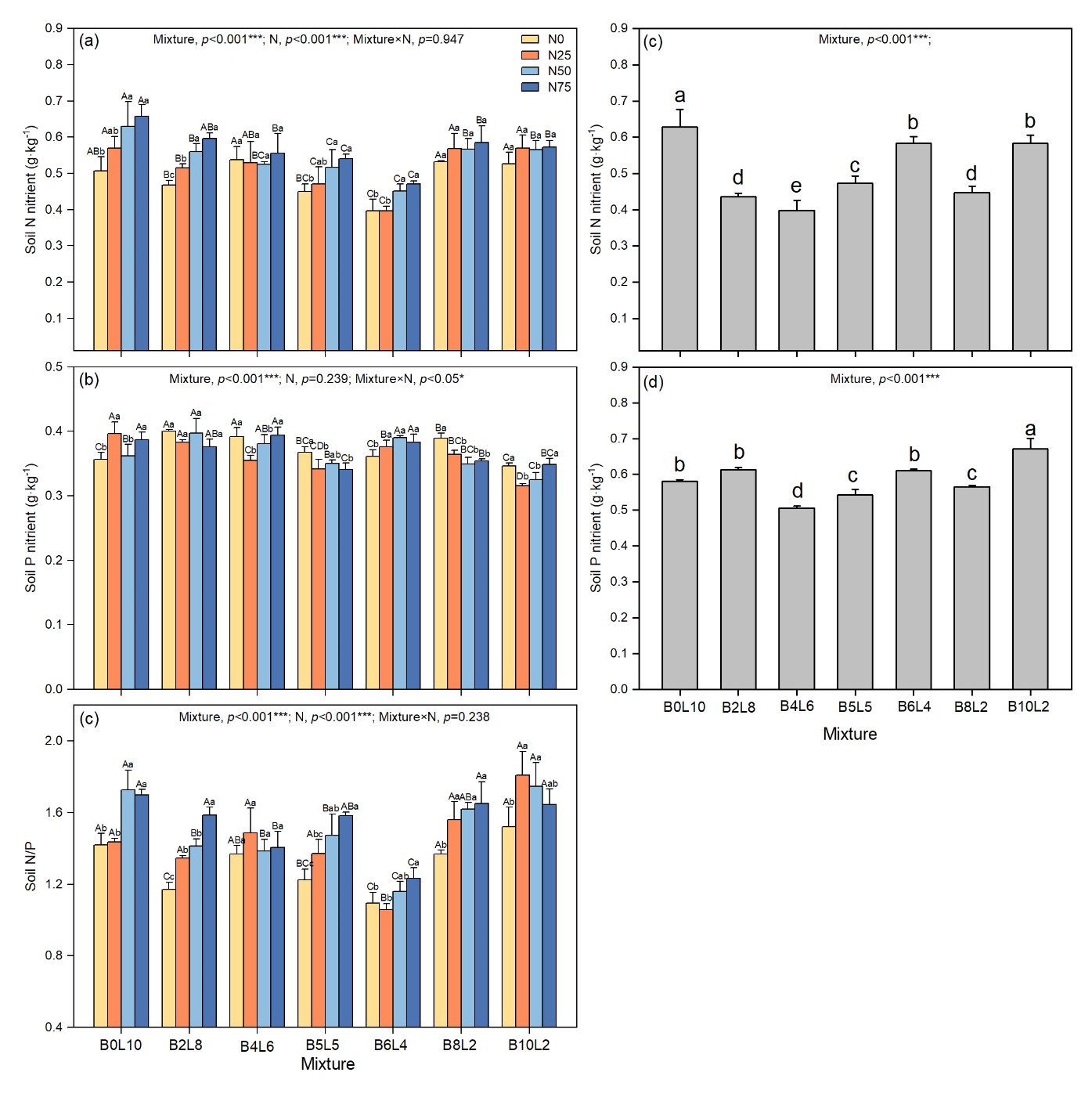


**Table S1** **Results of covariance analysis of the effects of mixture and N addition in fluorescence parameters when considering soil background values as covariates**

|  | ***B.ischaemum*** | | ***L.davurica*** | |
| --- | --- | --- | --- | --- |
|  | *F* | *p* | *F* | *p* |
| **Fo** | | | | |
| STN_2018_ | 0.534 | 0.467 | 0.077 | 0.782 |
| STP_2018_ | 7.234 | ***0.008***** | 9.471 | ***0.003***** |
| Mixture | 3.925 | ***0.001***** | 11.554 | ***0****** |
| N | 0.419 | 0.74 | 4.345 | ***0.006***** |
| Mixture*N | 3.086 | ***0****** | 4.84 | ***0****** |
| **Fv/Fm** | | | | |
| STN_2018_ | 2.183 | 0.142 | 1.217 | 0.272 |
| STP_2018_ | 3.174 | 0.078 | 0.057 | 0.811 |
| Mixture | 4.046 | ***0.001***** | 0.74 | 0.619 |
| N | 8.607 | ***<0.001****** | 3.12 | ***0.029**** |
| Mixture*N | 7.354 | ***<0.001****** | 8.306 | ***<0.001****** |
| **ΦPSII** | | | | |
| STN_2018_ | 0.683 | 0.41 | 12.372 | ***0.001***** |
| STP_2018_ | 0.006 | 0.937 | 1.422 | 0.236 |
| Mixture | 21.825 | ***<0.001****** | 10.71 | ***<0.001****** |
| N | 112.594 | ***<0.001****** | 1.847 | 0.143 |
| Mixture*N | 13.141 | ***<0.001****** | 4.62 | ***<0.001****** |
| **NPQ** | | | | |
| STN_2018_ | 2.724 | 0.102 | 2.311 | 0.131 |
| STP_2018_ | 5.718 | ***0.018**** | 3.236 | 0.075 |
| Mixture | 40.303 | ***<0.001****** | 8.494 | ***<0.001****** |
| N | 11.261 | ***<0.001****** | 42.701 | ***<0.001****** |
| Mixture*N | 16.817 | ***<0.001****** | 16.751 | ***<0.001****** |
| **qP** | | | | |
| STN_2018_ | 0.344 | 0.559 | 0.943 | 0.334 |
| STP_2018_ | 0.249 | 0.619 | 7.239 | ***0.008***** |
| Mixture | 19.156 | ***<0.001****** | 31.124 | ***<0.001****** |
| N | 143.918 | ***<0.001****** | 1.606 | 0.192 |
| Mixture*N | 10.607 | ***<0.001****** | 9.017 | ***<0.001****** |

**Table S2 Results of covariance analysis of the effects of mixture and N addition in fluorescence parameters when considering soil background values as covariates**

|  | **Aboveground biomass** | | **Relative yield (RY) for *B.ischaemum*** | | **Relative yield (RY) for *L.davurica*** | |
| --- | --- | --- | --- | --- | --- | --- |
|  | *F* | *p* | *F* | *p* | *F* | *p* |
| STN_2018_ | 1.769 | 0.186 | 0.937 | 0.339 | 0.856 | 0.361 |
| STP_2018_ | 2.017 | 0.158 | 0.988 | 0.327 | 0.009 | 0.925 |
| Mixture | 342.757 | ***<0.001****** | 16.008 | ***<0.001****** | 17.857 | ***<0.001****** |
| N | 333.524 | ***<0.001****** | 90.375 | ***<0.001****** | 147.156 | ***<0.001****** |
| Mixture*N | 60.932 | ***<0.001****** | 35.246 | ***<0.001****** | 21.166 | ***<0.001****** |

**Fig S2** Response of plant nutrient content, phosphorus content, and plant N:P ratio of *B.ischaemum* and *L.davurica* to mixture ratios and nitrogen additions.


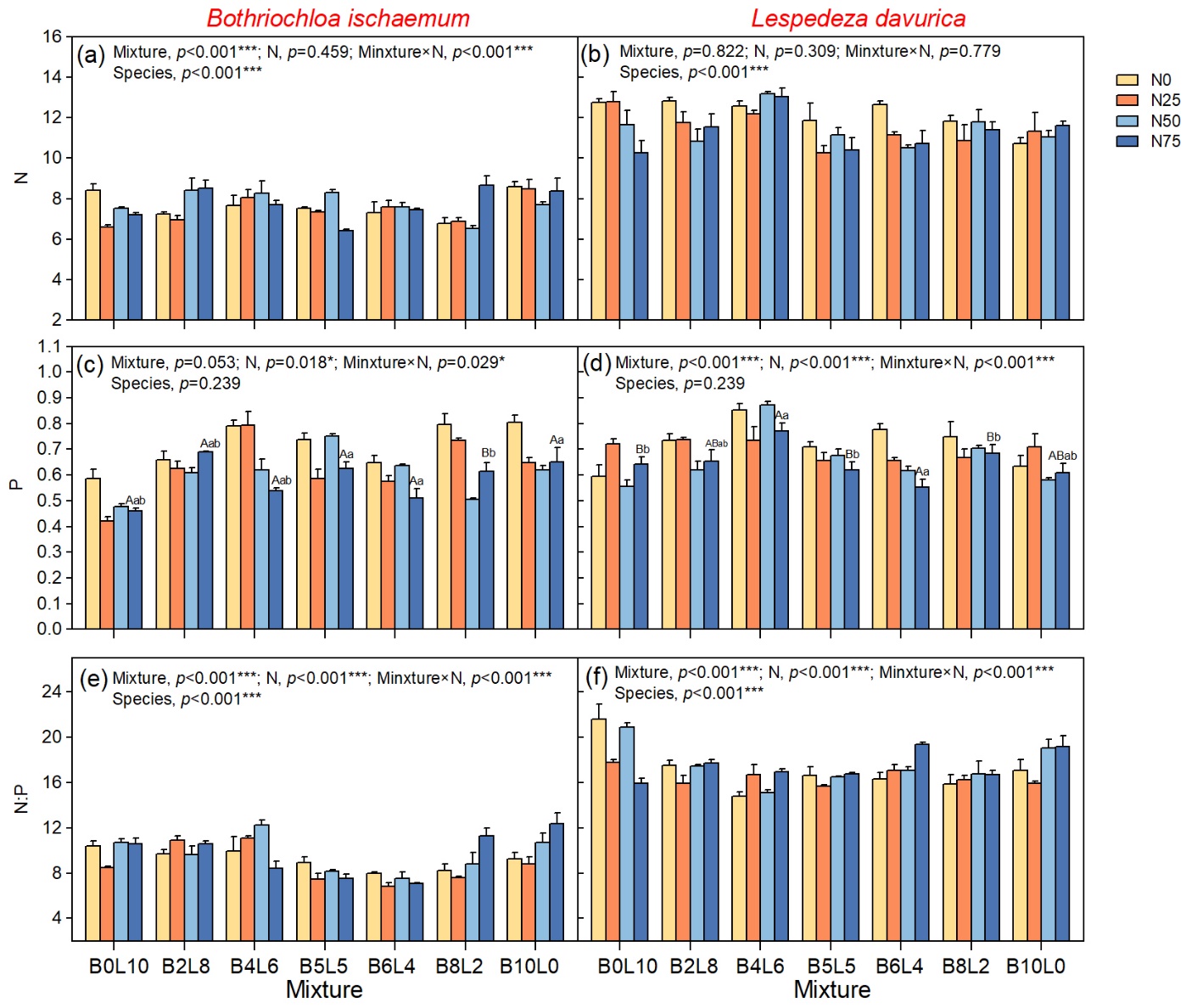


**Fig S3** The total effects, direct and indirect effects of factors paths of PLS-PM.


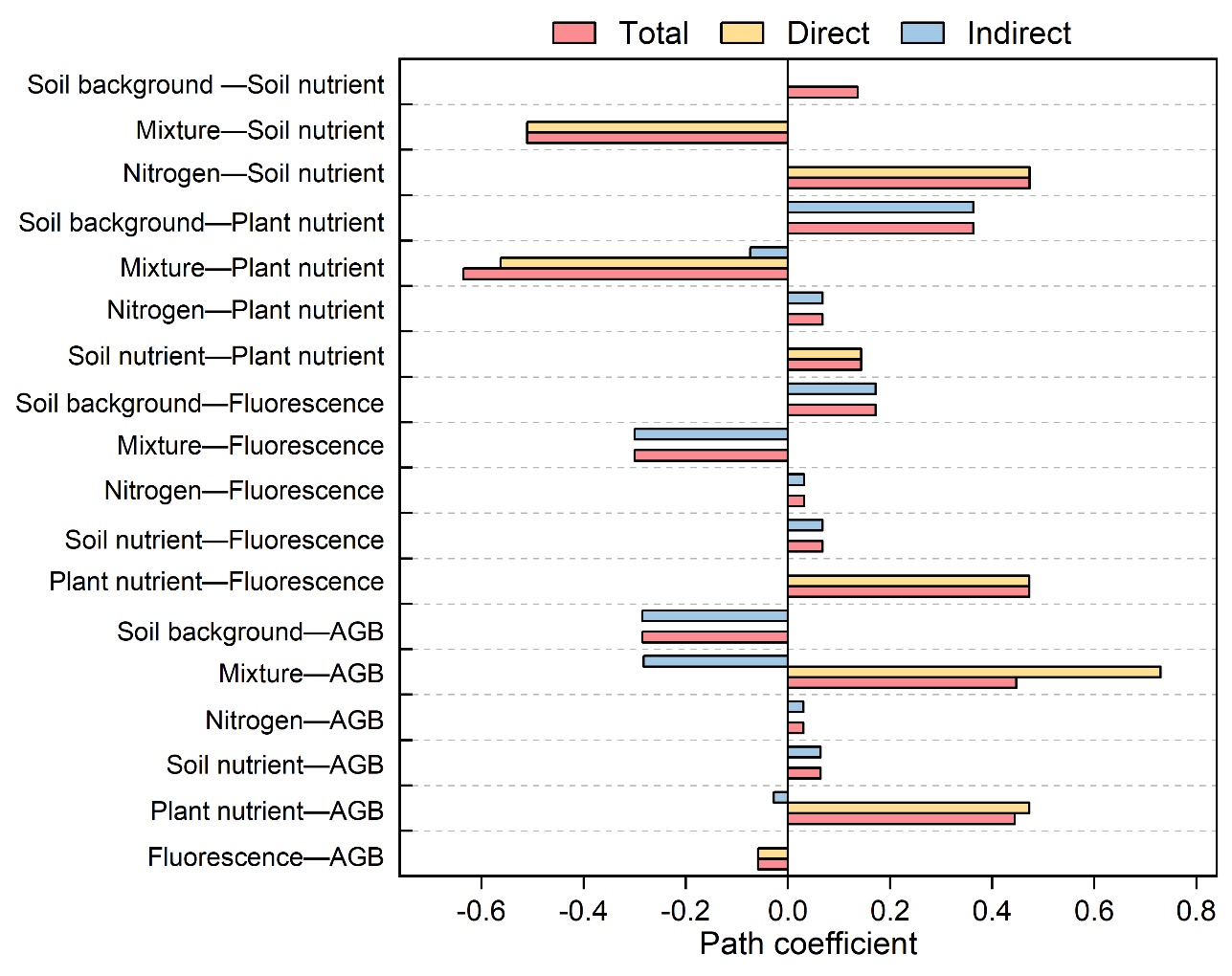

Supplement: Supplementary file 1 [file DataSheet_1.docx]
